# Probiotics as Beneficial Dietary Supplements to Prevent and Treat Cardiovascular Diseases: Uncovering Their Impact on Oxidative Stress

**DOI:** 10.1155/2019/3086270

**Published:** 2019-05-07

**Authors:** Elisardo C. Vasquez, Thiago M. C. Pereira, Veronica A. Peotta, Marcelo P. Baldo, Manuel Campos-Toimil

**Affiliations:** ^1^Laboratory of Translational Physiology and Pharmacology, Pharmaceutical Sciences Graduate Program, Vila Velha University (UVV), Vila Velha, Brazil; ^2^Federal Institute of Education, Science and Technology (IFES), Vila Velha, Brazil; ^3^Stead Family Department of Pediatrics, The University of Iowa, Iowa City, IA, USA; ^4^Department of Pathophysiology, State University of Montes Claros, Montes Claros, MG, Brazil; ^5^Department of Medicine, Faculdades Integradas Pitagoras, Montes Claros, MG, Brazil; ^6^Pharmacology of Chronic Diseases (CD Pharma), Center for Research in Molecular Medicine and Chronic Diseases (CIMUS), University of Santiago de Compostela, Santiago de Compostela, Spain

## Abstract

The gut microbiota, the ecosystem formed by a wide symbiotic community of nonpathogenic microorganisms that are present in the distal part of the human gut, plays a prominent role in the normal physiology of the organism. The gut microbiota's imbalance, gut dysbiosis, is directly related to the origin of various processes of acute or chronic dysfunction in the host. Therefore, the ability to intervene in the gut microbiota is now emerging as a possible tactic for therapeutic intervention in various diseases. From this perspective, evidence is growing that a functional dietary intervention with probiotics, which maintain or restore beneficial bacteria of the digestive tract, represents a promising therapeutic strategy for interventions in cardiovascular diseases and also reduces the risk of their occurrence. In the present work, we review the importance of maintaining the balance of the intestinal microbiota to prevent or combat such processes as arterial hypertension or endothelial dysfunction, which underlie many cardiovascular disorders. We also review how the consumption of probiotics can improve autonomic control of cardiovascular function and provide beneficial effects in patients with heart failure. Among the known effects of probiotics is their ability to decrease the generation of reactive oxygen species and, therefore, reduce oxidative stress. Therefore, in this review, we specifically focus on this antioxidant capacity and its relationship with the beneficial cardiovascular effects described for probiotics.

## 1. Introduction

Literature searches on “gut microbiota” performed by the guest editors via the PubMed platform revealed extensive publications, consisting of approximately 880 reviews in 2018 (~2/day). Approximately 380 (~1/day) reviews on “probiotics” were published in 2018. Restricting the search with filters to published reviews using the combination of terms “probiotics and cardiovascular diseases” reduced the number to 15. Interestingly, when the term “oxidative stress” was included in the combination “probiotics and cardiovascular diseases (CVD) and oxidative stress,” no published reviews were identified. It is known that CVD remains the leading cause of death and disability in developed countries. These two scenarios associated with exciting original articles on probiotics and CVD in the prior 3 years motivated us to promote the publication of this special issue.

**Figure 1:**
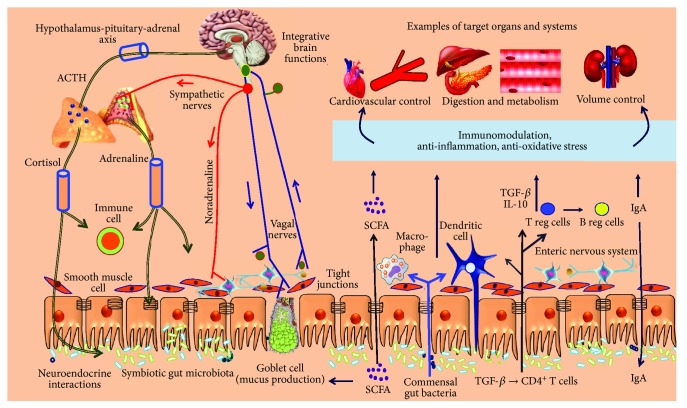
Summary of the interaction between the gut microbiota and neuroimmune and neuroendocrine systems and the interaction between the gut microbiota and microbiota target organs of the host. The gut microbiota provides (i) a mucosal barrier through tight and adherens junctions between enterocytes, (ii) immunomodulation and anti-inflammation through recruitment of immune cells, and (iii) energy metabolism via metabolites/short-chain fatty acids (SCFAs), vitamins, and hormones. The brain-intestine axis acts through both an integrative autonomic nervous system, including the sympathetic/parasympathetic (vagal) afferent/efferent nerve pathways, associated with the neural myenteric network, and a neuroendocrine system, including the hypothalamus-adrenocortical gland system.

The gut microbiota is a broad symbiotic community of nonpathogenic microorganisms composed primarily of anaerobic bacteria (although some gut bacteria preferentially grow under microaerophilic conditions) and fungi [1]. One of its functions is the maintenance of a barrier via enterocytes covered with a brush border of mucus, which is produced by goblet cells and nonpenetrable tight junctions between enterocytes [2, 3], a layer of luminal mucus and tight adherens junctions between enterocytes that allows the control of absorption and metabolism and the maturation and stimulation of the immune system, which are essential functions for an effective mechanism of defense against pathogens in the host [4]. Signals generated in the gut microbiota communicate with distant organs by crossing the intestinal epithelium and triggering diverse signaling processes located at the epithelial cell border, subsequently reaching the systemic circulatory system. Different pathways are responsible for the bidirectional interaction between the gut microbiota and systemic organs in healthy individuals (Figure 1).

Currently, there is a growing body of evidence that an abnormal predominance of pathogenic over commensal (nonpathogenic) microorganisms, a condition termed gut dysbiosis, can initiate or worsen the dysfunction of diverse target systemic organs [5–7]. Gut dysbiosis is also a confirmed cause of increased oxidative stress in the body. In fact, frequent consumption of fats and refined sugars in the Western-type diet produces an increase in reactive oxygen species (ROS) production and inflammatory processes [8, 9]. Additionally, the gut microbiota regulates the production of mitochondrial ROS [10]. In recent years, various studies have revealed that gut dysbiosis may contribute to the development and progression of CVD and other related diseases [11, 12].

On the other hand, several studies have demonstrated that probiotics may be beneficial in reestablishing the microbiota through different mechanisms such as appropriate intestinal homeostasis [13]. The name probiotic is applied to those live microorganisms which, when administered in adequate amounts, confer a health benefit on the host. This definition, established by the joint Food and Agriculture Organization of the United Nations (FAO) and World Health Organization (WHO) Working Group, is the one used today and accepted by the International Scientific Association for Probiotics and Prebiotics (ISAPP) [14].

Therefore, the purpose of this review is to update our knowledge concerning the contribution of the exacerbated production of oxidative stress to the development of cardiac and vascular dysfunctions in the clinic and in experimental models of arterial hypertension, as well as the possible beneficial effects of dietary supplementation with probiotics, in an attempt to prevent or reverse these cardiovascular disturbances.

## 2. Probiotics as Promising Coadjuvants for Prevention/Treatment of Arterial Hypertension

Arterial hypertension constitutes a main risk factor for the development of severe pathologies, such as acute myocardial infarction, heart failure, stroke, and renal failure [15–17], as well as for premature death worldwide [18]. Primary (or essential) hypertension is a multifactorial process that involves genetics, demographics, comorbid disorders, and environmental influences [19]. Approximately 8% of cases exhibit secondary hypertension, which has a known origin, including endocrine diseases, drugs, cancer, or hyperactivation of the renin-angiotensin system, among others [20].

Antihypertensive therapy used in clinical practice has been shown to be effective in maintaining blood pressure (BP) at safer levels, thereby reducing the morbidity and mortality associated with this disease. Several international reports, mainly those prepared by the Eighth Joint National Committee (JNC 8), have established the guidelines for the treatment of hypertensive patients and different reference values for those over 60 years old [21].

In addition to pharmacological treatments, it is necessary to establish a series of nonpharmacological measures for the control of the disease. In this sense, the relevance of a good diet is paramount [22], and the contribution of probiotics can be fundamental. It has been demonstrated that the gut microbiota participates in an important manner in the control of BP by several mechanisms, such as exerting control at the level of the central and autonomic nervous system or protecting endothelial function (see illustration in Figure 1). Additionally, gut dysbiosis has been described in animal models of hypertension and hypertensive patients [23].

In agreement with the above discussion and based on the state-of-the-art role of the gut microbiota and its interaction with different organs, a working group at the National Heart, Lung, and Blood Institute recently discussed the current status and future directions for the treatment and prevention of high BP, considering the use of probiotics [24]. Therefore, functional foods that contribute to the maintenance of the intestinal flora can be very useful when avoiding excessively high BP levels, as previously reviewed in detail by several authors (see [11, 25–28]).

**Figure 2:**
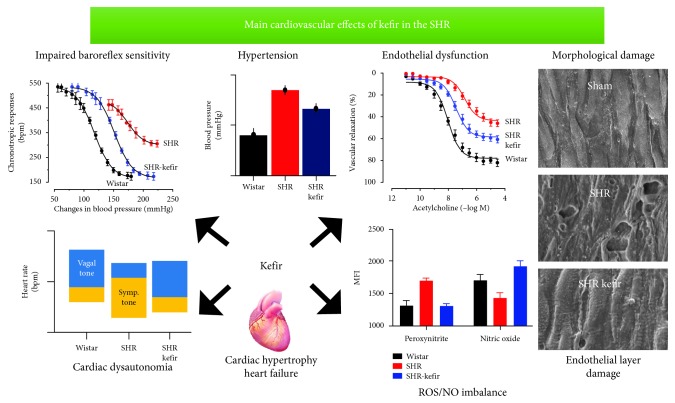
Main cardiovascular disturbances observed by our research group in the SHR model and the effectiveness of kefir supplementation to attenuate or revert them. Graphs were constructed based on published data [28–30, 32].

Among the foods that have been shown to provide various cardiovascular benefits, kefir has been reported to effectively lower BP [5, 28]. Chronic consumption of this synbiotic attenuates the abnormal increase in BP in spontaneously hypertensive rats (SHR), which has been the most commonly used genetically hypertensive animal for a better understanding of several cardiovascular abnormalities. Kefir has been tested in the protection of vascular endothelial dysfunction [29] and in the correction of impaired autonomic cardiovascular function [30, 31], including its inhibitory effects on angiotensin-converting enzyme (ACE) [32] (Figure 2). Therefore, there is growing evidence that probiotics could be a promising natural coadjuvant in the prevention/treatment of CVD, including the hypertensive process. In another study using SHR, animals fed with Minas Frescal probiotic cheese showed a significantly lower BP compared with the control group, in addition to an improvement in other indicators of cardiovascular health, such as blood levels of triglycerides and cholesterol [33].

Several clinical studies in humans have also demonstrated the ability of probiotics to reduce abnormally high BP levels. For example, an extract of *Lactobacillus casei*, which has been shown to reduce BP in SHR [34], was able to induce a reduction in systolic/diastolic BP and heart rate in hypertensive patients [35]. In 2002, an interesting study showed that food supplementation with *Lactobacillus plantarum* produced a significant decrease in systolic BP in heavy smokers [36]. A Norwegian study showed, in 2011, that the incidence of preeclampsia, which is associated with hypertension and inflammation, is decreased by chronic intake of probiotics [37]. Additionally, in a randomized double-blind clinical trial with type II diabetes mellitus, probiotic soy milk containing *Lactobacillus plantarum* significantly decreased systolic/diastolic BP [38], and in a study with prediabetic patients, there was a significant tendency to reduce hypertension in those patients receiving a multispecies probiotic [39]. In 2014, a meta-analysis carried out based on the results of nine clinical trials found that consumption of probiotics slightly reduced BP and that this effect was more marked if the basal BP was elevated. The authors also concluded that several species of probiotics used together provided enhanced effects. Finally, the duration of the intervention must be ≥8 weeks, and the dose of daily consumption of probiotics should be ≥10^11^ colony-forming units [40].

In contrast, several studies have questioned the role of some probiotics in producing low BP. A treatment for 4 weeks by dietary supplementation of *Lactobacillus plantarum* either together with fermented blueberry or with three synthesized phenolic compounds did not lower BP in NG-nitro-L-arginine methyl ester- (L-NAME-) induced hypertensive rats [41]. In a clinical trial, probiotic strains of *Lactobacillus acidophilus* and *Bifidobacterium animalis*, provided in either yogurt or capsule form, did not improve cardiovascular risk factors since they did not modify BP or concentrations of total cholesterol LDL-C, HDL-C, or triglycerides in overweight or obese individuals [42]. Additionally, a study of postmenopausal women with metabolic syndrome showed that administration of milk supplemented with *Lactobacillus plantarum* produced several beneficial effects, but it did not provide a significant decrease in BP [43]. Furthermore, long-term treatment with *Lactobacillus helveticus-*fermented milk containing bioactive peptides reduced arterial stiffness in hypertensive subjects but did not induce statistically significant differences between the effects of the probiotic and placebo treatment on BP [44].

In view of these reports, it will still be necessary to carry out more studies to verify the possible role of probiotic foods as coadjuvants in the treatment of arterial hypertension. In any case, the results of the different studies suggest that the complicated mechanisms of the development of hypertension, the choice of different bacterial strains, the different types of patients, and the previous state of their microbiome can be decisive in terms of obtaining satisfactory results for the reduction of BP.

## 3. Endothelial Dysfunction: The Role Played by Oxidative Stress

The vascular endothelium is a single layer of smooth, thin cells that constitutes the first barrier between the bloodstream and the vascular muscle. Among its functions is to act as a selective membrane through which fluid and solutes, as well as trafficking of inflammatory cells, interchange between the plasma and tissue spaces [45, 46].

The endothelium also contributes to the regulation of vascular tone by synthesizing and releasing a huge number of vasodilating substances, both vasodilators such as nitric oxide (NO), prostacyclin, and endothelium-derived hyperpolarizing factor (EDHF) and vasoconstrictors such as endothelin (via ET_A_), angiotensin II (via AT_1_ receptors), and ROS. In addition, its action is the key in the control of platelet aggregation and blood hemostasis, regulating the antithrombotic/prothrombotic balance, and it also participates in the inflammatory and immune response (for a detailed review, see [47–49]).

Due to this multifunctional role of the endothelium, it is easy to understand that its alterations may lie at the origin and/or in the development of various diseases. Therefore, endothelial dysfunction is recognized as a risk factor for the onset of CVD and appears in the early stages and during the development of hypertension, cardiac ischemia, atherosclerosis, stroke, or peripheral vascular disease [48, 50, 51]. Other diseases such as diabetes, kidney failure, infectious diseases, and tumor progression also have a component of endothelial dysfunction [49, 51, 52].

Endothelial dysfunction can be caused by inflammatory processes, leading to a decrease in endothelial NO synthase (eNOS) enzyme activity, thereby decreasing the NO bioavailability and culminating in hypertension [53]. Moreover, oxidative stress also contributes to the development of endothelial dysfunction, reducing the availability of NO [54, 55]. In fact, the generation of ROS caused by hypertension, hypercholesterolemia, diabetes, or other cardiovascular risk factors causes a decrease in the release of endothelial NO [56].

As mentioned above, there is an important relationship between dysbiosis and the development of hypertension (see also [57]), which could involve the impaired endothelial function due to alterations of the gut microbiota during the chronic rise in BP. In fact, fecal microbiota transplantation from SHR to normotensive WKY rats caused a chronic impairment of endothelial function, accompanied by greater vascular oxidative stress and increased systolic BP. In contrast, transplantation of fecal microbiota from WKY to SHR provoked the opposite effects with an improvement of endothelial dysfunction in hypertensive animals [27].

Accordingly, several studies have suggested that probiotics could lead to an improvement in endothelial function. Rashid et al. [58] reported that the endothelial dysfunction of mesenteric artery rings in rats with common bile duct ligation is mediated in part by oxidative stress, possibly due to bacterial translocation inducing a proinflammatory response, and that this effect is improved by the ingestion of a probiotic formulation.

**Figure 3:**
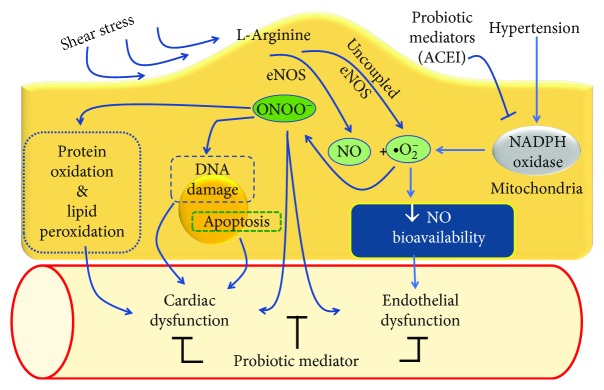
Main mechanisms of action of probiotics on the endothelial layer of conductance and resistance vessels showing the deleterious actions of reactive oxygen species and the beneficial actions of probiotics, leading to the attenuation of endothelial dysfunction observed in hypertensive subjects.

Endothelial dysfunction can be identified physiologically by means of NO-dependent mechanisms (Figure 3). In this situation, blood vessels show a reduced vasodilator response to agents that contribute to the release of NO, such as acetylcholine and, conversely, an exacerbated response to vasoconstrictor agents, such as *α*_1_-adrenergic agonists or thromboxane A2 analogues. Using this method, chronic probiotic treatment with *Lactobacillus coryniformis* reversed the endothelial dysfunction observed in obese mice and improved the endothelial dysfunction and vascular oxidative stress induced by lipopolysaccharides (LPS) in control mice [59].

In a similar way, our group evaluated the effects of the probiotic kefir on endothelial dysfunction in SHR. Our results suggested that kefir treatment for eight weeks (even at a low dose) could attenuate endothelial dysfunction in the large vessels in hypertensive rats, and the main mechanism for this beneficial effect was exerted through a repair of the vascular endothelial architecture (Figure 2) and a reduction of the oxidative stress, together with an increase in NO bioavailability as well as endothelial progenitor cell recruitment [29]. These beneficial effects of kefir on vascular endothelial function have recently been reviewed [5].

This effect of probiotics was also confirmed in a study in which lactic acid bacteria partially reversed the relaxation deficit of the aorta in SHR. In addition, it also increased the NO level, which is abnormally decreased in SHR serum. Both effects are indicative of a probiotic-induced improvement in endothelial function due to a reduction of vascular oxidative and inflammatory status [60].

In addition, using SHR and WKY rats for comparison, a study by Gomez-Guzman et al. [61] demonstrated that chronic oral administration of the probiotic *Lactobacillus fermentum* or *Lactobacillus coryniformis* plus *Lactobacillus gasseri* restored gut eubiosis and improved endothelial dysfunction as a result of a reduced vascular proinflammatory and prooxidative status.

Some studies in humans or human cells have also shown an improvement in endothelial function due to probiotic treatment. In endothelial cells, soy milk fermented with *Lactobacillus plantarum* or *Streptococcus thermophilus* stimulated NO production and eNOS activity, suggesting their effectiveness for the improvement of endothelial function [62]. A 6-week supplementation with *Lactobacillus plantarum* in men with stable coronary artery disease improved endothelial function for both conduit and resistance vessels through increasing NO bioavailability while concomitantly reducing systemic inflammation, as measured by brachial artery flow-mediated dilation. These results suggest that the intestinal microbiota is mechanistically linked to systemic inflammation and vascular endothelial function [45]. Another clinical trial showed that a multispecies probiotic supplement improved both functional and biochemical parameters of endothelial dysfunction, including systolic BP, vascular endothelial growth factor, pulse wave velocity (PWV) and its augmentation index, interleukin-6, tumor necrosis factor alpha (TNF*α*), and thrombomodulin in obese postmenopausal women [63]. In contrast, in a study of subjects with metabolic syndrome receiving supplementation with the probiotic strain *Lactobacillus casei Shirota*, no significant changes in parameters used to assess low-grade inflammation or endothelial dysfunction were observed [64].

In general, studies both *in vivo* and *in vitro*, as well as clinical studies in humans, suggest that supplementation with several types of probiotics contributes to an improvement in endothelial function through various mechanisms. Although further research is needed, the role of probiotic supplementation in the prevention of CVD by correcting endothelial dysfunction is promising. In addition, the multifunctional role of the endothelium extends this potential use of probiotics to all diseases, not only cardiovascular, in which its pathophysiology may be related to endothelial dysfunction.

## 4. Evidence of the Beneficial Effects of Probiotics on the Autonomic Control of Cardiovascular Function

Prebiotics, probiotics, and synbiotics are some of the best evidenced ways of manipulating the microbiota, and their potential role in the prevention and treatment of multiple diseases has recently garnered a significant interest. Recent data from experimental time-course studies have shown that long-term treatment with *kefir* (at least 30 to 60 days), in addition to the antihypertensive effect, attenuated cardiac hypertrophy in SHR [29, 30, 32]. Considering the relationship between the gut microbiota and the target systemic organs, it is important to highlight studies that relate the influence of these microorganisms to cardiovascular function.

**Figure 4:**
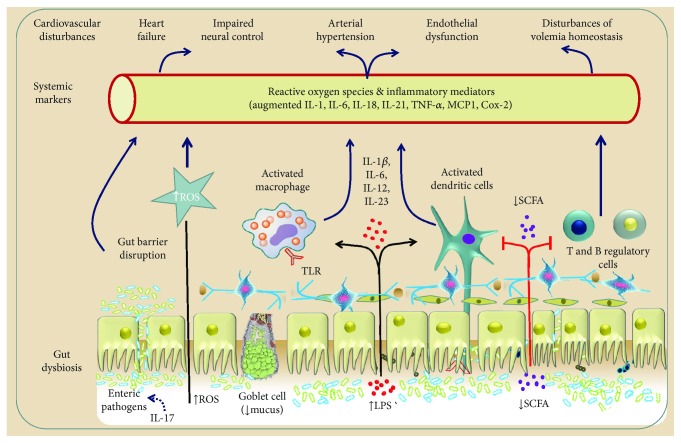
Gut microbiota and some relevant molecular pathways linking gut dysbiosis to cardiovascular and cardiometabolic diseases through the bloodstream and via the autonomic nervous system. The main mechanisms of the modulators include the neuroendocrine hypothalamus-pituitary-adrenal axis (ACTH: adrenocorticotrophic hormone), afferent and efferent pathways of the autonomic nervous system (vagal and sympathetic components), reactive oxygen species (ROS), inflammatory markers (interleukins: IL; tumor necrosis factor *α*: TNF*α*; monocyte chemoattractant protein: MCP1; cyclooxygenase: Cox 2; toll-like receptors: TLR), and dietary metabolic byproducts (short-chain fatty acids: SCFAs; lipopolysaccharides: LPS).

Those findings led the authors to investigate whether the benefits of kefir supplementation could also include the autonomic neural control of BP (baroreflex function) and the cardiac pacemakers controlling the chronotropic rhythm under the neural efferent pathways from the brainstem integrative areas. They observed that administration of kefir (for at least 60 days) attenuated and partially reversed the abnormal cardiac sympathetic predominance over the parasympathetic tone in SHR, raising the following question: “by which mechanisms can probiotics and synbiotics affect brain areas?” As illustrated in Figures 1 and 4, there is a consistent and well-recognized neuroendocrine gut-brain axis connection, which includes the hypothalamus-pituitary-suprarenal gland axis and the autonomic sympathetic/parasympathetic afferent/efferent pathways. Others have attempted to address the question by proposing relevant interactions between gut endocrine cells and vagal afferents through gut chemosensing mechanisms [65, 66].

Other exciting findings in recent years has been the demonstration of a marked association between the effects of probiotics and decreased production of intravascular ROS and augmented NO bioavailability. It seems that the mechanisms underlying the beneficial actions of probiotics on cardiac autonomic control could occur through its capability to decrease the production of cytokines and ROS in the hypothalamic paraventricular nucleus. In turn, it could attenuate hypertension and end-organ damage by upregulating anti-inflammatory and antioxidant molecules, therefore restoring the normal balance between parasympathetic and sympathetic activity to the heart, as recently observed by our group [30]. In a similar way and as expected, the investigators observed that SHR treated with probiotics presented a partial recovery of the baroreceptor sensitivity, which is characterized in this experimental model by a high variability of the resting BP. The SHR exhibited diminished reflex tachycardia or bradycardia to induce hypotensive or hypertensive changes in the resting BP. Probiotic supplementation was able to partially repair this BP variability and baroreflex sensitivity, and this could occur because kefir repairs the normal gut microbiota and, consequently, restores the production of neuroactive compounds in the intestinal lumen. Therefore, the above findings corroborate the knowledge that probiotics have a modulatory action on the integrative central or peripheral components of the gut-brain axis [65, 67].

Recently, Brasil et al. [32] assessed whether the soluble nonbacterial fraction of kefir (bioactive compounds) and not the probiotic effects would improve cardiovascular hemodynamics, enhancing the baroreflex sensitivity, which could include the ACE inhibitory properties. Therefore, an important mechanism by which probiotics decrease high BP and repair endothelial dysfunction and cardiac autonomic tones could be achieved through probiotic bioactive compounds. In addition, it has been observed that probiotic supplementation caused a decrease in ACE activity measured in the serum of SHR treated with the soluble nonbacterial fraction of kefir, supporting ACE inhibition as a likely mechanism for kefir's beneficial cardiovascular effects during hypertension. These effects indicate that the improvement in baroreflex gain cannot be attributed to the probiotic effect of kefir but rather to other bioactive compounds produced by microbial action. Clearly, how these different fractions (e.g., probiotic bacteria or bioactive compounds) influence the baroreflex and other cardiovascular risk factors are still poorly understood. To our knowledge, very few publications have evaluated the effects of probiotics on baroreflex function and autonomic control of heart rate. These previous studies used the fermented food *kefir* [30] and its bioactive compounds [32].

**Figure 5:**
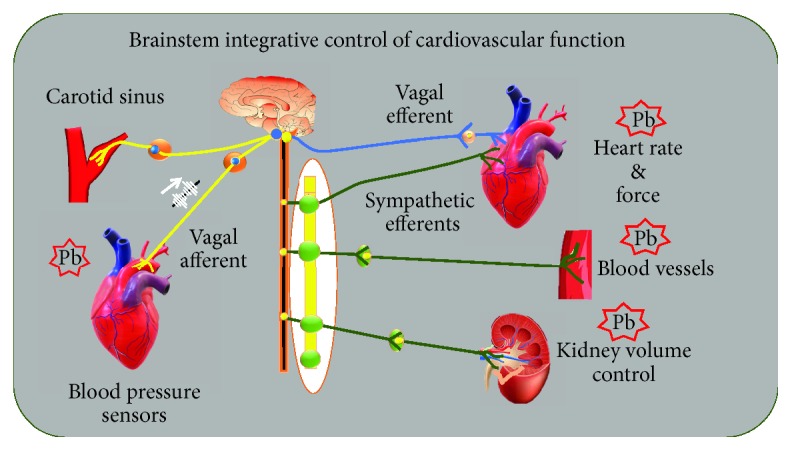
Diagram illustrating the pathways of neural control of cardiac and vascular function through the autonomic neural system and possible sites of action of the probiotics (Pb) based on recent evidence. Mechanosensitive baroreceptors are located in the carotid sinus and aortic arch, and they discharge at BP systole-by-systole bursts of action potentials to the brainstem. The autonomic vagal and sympathetic efferents innervate the cardiac pacemakers and the myocardium (sympathetic ends). The resistance and conductance vessels are innervated by sympathetic efferent components. Modified from Vasquez et al. [28], with permission.

In conclusion, the benefits of probiotics in the cardiovascular system in models of hypertension include the reversion of cardiac dysautonomia, which is characterized in hypertensive subjects by an inverted predominance of sympathetic over vagal tone, including a significant attenuation of the high variability of BP and heart rate and their effectiveness to partially revert the decreased [68] baroreflex sensitivity. Nonetheless, probiotics attenuate disturbances in the neural control of cardiovascular function in a similar manner to that achieved with physical exercise [68], therapy with flavonoids [69], and pharmacological medication [30]. Therefore, there is clearly a need for more mechanistic studies that would help to identify the missing links to explain the protective effects of fermented foods, such as pre-, pro-, and synbiotics, as well as their bioactive compounds on the neural control of BP.  Figure 5 summarizes the possible sites of action of probiotics.

## 5. Heart Failure: A Target for the Benefits of Functional Diets

Heart failure (HF) patients experience some changes in the gut microbiome during disease. Some reports have described increased levels of pathogenic microbes that could have potential deleterious effects on cardiac function [70]. This phenomenon might be explained by the so-called “gut hypothesis,” in which the reduced cardiac pumping function and congestion observed in HF patients would be responsible for an intestinal ischemia [71], favoring bacterial translocation and increases in circulating endotoxins that elicit inflammation [72]. In fact, the intestinal blood flow is reduced in HF patients, contributing to juxtamucosal bacterial growth [73]. Kummen et al. [74] reported that the gut microbiota in HF patients is related to persistent T-cell activation. In fact, the removal of Gram-negative intestinal bacteria by antibiotics reduces the monocyte CD14 expression, along with reduced levels of endotoxins and cytokines, with improved flow-mediated dilation in patients with severe HF [75].

The association between gut dysbiosis and CVD was highlighted when 60 stable HF patients were selected to test whether the characteristics of the gut microbiota would correlate with their cardiovascular functional status. The authors evidenced that HF patients had more colonies of pathogenic bacteria than control participants, along with an increased intestinal permeability that favored bacterial translocation. In addition, severe HF was associated with more pathogenic types of bacteria than mild HF [76]. The contribution of the gut microbiota to the pathogenesis of CVD has been supported by the discovery that some dietary products that are metabolized by gut microbes produce toxic metabolites that could have negative impact on the cardiovascular system. Changes in the gut microbiota can lead to increases in trimethylamine N-oxide (TMAO), which is a major contributor to cardiovascular and renal diseases [77]. TMAO is an endotoxin that is produced via the metabolism of trimethylamine from the carnitine molecule, which is absorbed into the blood and converted into TMAO in the liver by flavin-containing monooxygenases [78]. In fact, intestinal microbes participate in the phosphatidylcholine metabolism and in the increased TMAO levels, which was independently associated with major cardiovascular events [70] and the incidence of chronic kidney diseases [79]. The findings also showed that TMAO could contribute to the risk prediction scores of deaths in acute HF patients, revealing a poor 1-year prognosis [80].

Experimental studies have demonstrated potential therapeutic actions of probiotics in different animal models of HF. Using a rat model of acute myocardial infarction by permanent coronary occlusion, Gan et al. [81] showed improved ventricular function and structure after treatment with the probiotic *Lactobacillus rhamnosus*. The possible mechanism by which probiotics act in the infarcted heart has been described by Lam et al. [82], in which the probiotic *Lactobacillus plantarum* decreased the leptin levels and, thus, reduced the infarct size in rats. Additionally, the antiapoptotic effect of probiotic-fermented purple sweet potato yogurt was evidenced in a rat model of hypertensive HF [83]. Despite the increasing number of experimental studies on probiotics in HF models, only one study has addressed the prognostic effects of probiotics in HF patients. In this pilot trial, patients with HF class II or III and LVEF <50% were randomized to *Saccharomyces boulardii* or placebo for 3 months in a double-blinded fashion. Patients treated with the probiotic showed a significant reduction in the left atrial diameter, uric acid, CRP, and creatinine levels. An important find of this study was that the treatment was safe and well tolerated, without reports of side effects or adverse events [84].

## 6. Conclusions

Throughout this review, we have presented evidence in the literature indicating that a habitual consumption of probiotics, which restore the balance of the intestinal microbiota, could present cardiovascular benefits based, at least in part, on its ability to reduce oxidative stress. Although many points remain to be clarified and many of the published results are contradictory, it is evident that consumption of probiotics constitutes a promising complement to more conventional cardiovascular therapies, as well as to nonpharmacological measures that are commonly used to counteract the onset and progression of CVD. Further studies are needed to clarify the interaction between the gut microbiota and the neuroimmune system, as well as the endocrine system, to create nutrigenetic profiles that may aid in achieving individual homeostasis. It will also be necessary to improve knowledge concerning the different bacterial strains present in probiotics and how they should be consumed to take full advantage of their potential beneficial effects for each specific situation. Finally, studies of the great variety of enzymes, peptides, and biochemical pathways generated by the intestinal microbiota, which differ from the resources of the host, could constitute an innovative strategy for the design of new drugs for the treatment of CVD.
